# Cryo-EM structures of RNA polymerase II–nucleosome complexes rewrapping transcribed DNA

**DOI:** 10.1016/j.jbc.2023.105477

**Published:** 2023-11-17

**Authors:** Munetaka Akatsu, Haruhiko Ehara, Tomoya Kujirai, Risa Fujita, Tomoko Ito, Ken Osumi, Mitsuo Ogasawara, Yoshimasa Takizawa, Shun-ichi Sekine, Hitoshi Kurumizaka

**Affiliations:** 1 Laboratory of Chromatin Structure and Function, Institute for Quantitative Biosciences, The University of Tokyo, Bunkyo, Tokyo, Japan; 2 Department of Biological Sciences, Graduate School of Science, The University of Tokyo, Bunkyo, Tokyo, Japan; 3 Laboratory for Transcription Structural Biology, RIKEN Center for Biosystems Dynamics Research, Yokohama, Japan

**Keywords:** RNA polymerase, nucleosome, chromatin, cryo-electron microscopy, transcription, DNA looping

## Abstract

RNA polymerase II (RNAPII) transcribes DNA wrapped in the nucleosome by stepwise pausing, especially at nucleosomal superhelical locations −5 and −1 [SHL(-5) and SHL(-1), respectively]. In the present study, we performed cryo-electron microscopy analyses of RNAPII–nucleosome complexes paused at a major nucleosomal pausing site, SHL(-1). We determined two previously undetected structures, in which the transcribed DNA behind RNAPII is sharply kinked at the RNAPII exit tunnel and rewrapped around the nucleosomal histones in front of RNAPII by DNA looping. This DNA kink shifts the DNA orientation toward the nucleosome, and the transcribed DNA region interacts with basic amino acid residues of histones H2A, H2B, and H3 exposed by the RNAPII-mediated nucleosomal DNA peeling. The DNA loop structure was not observed in the presence of the transcription elongation factors Spt4/5 and Elf1. These RNAPII-nucleosome structures provide important information for understanding the functional relevance of DNA looping during transcription elongation in the nucleosome.

In eukaryotes, genomic DNA is compacted as chromatin. The basic unit of chromatin is the nucleosome, comprising approximately 150 to 200 base pairs of DNA and a histone octamer containing two each of histones H2A, H2B, H3, and H4 (1, 2). In chromatin, nucleosomes are connected by linker DNA segments and form a beads-on-a-string configuration (3).

In cells, transcription must occur on the DNA spooled in the nucleosome. During nucleosome transcription, RNA polymerase II (RNAPII) gradually peels the DNA from the histone surface without histone dissociation, until it reaches the center of the nucleosomal DNA (the dyad DNA) (4, 5). Previous studies with a prokaryotic RNA polymerase (RNAP) revealed that the nucleosomal histones in front of RNAP are dissociated when it passes through the dyad DNA of the nucleosome (6, 7). Histones are then transferred from in front to behind the transcribing RNAP, and the nucleosome is reassembled on the transcribed template DNA behind the RNAP (8). Histone transfer has also been observed during nucleosome transcription by eukaryotic RNAPII (9, 10, 11). This is consistent with the fact that the epigenetic information of the nucleosomes, such as histone post-translational modifications and histone variants, is maintained during transcription elongation.

Two mechanisms had been proposed for the RNAPII-dependent nucleosome transfer: the histone chaperone-dependent and -independent pathways (8, 12, 13, 14, 15, 16, 17). We previously reported the cryo-electron microscopy (cryo-EM) structures depicting the nucleosome disassembly and reassembly processes during RNAPII transcription in the histone chaperone-dependent pathway, in the presence of FACT, Spt4/5, Spt6, Spn1, Elf1, and Paf1C (18). Conversely, in the histone chaperone-independent pathway, the histone transfer from in front to behind RNAPII is proposed to occur *via* the formation of a template DNA loop, in which the upstream DNA exiting RNAPII rebinds to the histones within the partially unwrapped nucleosome downstream of the RNAPII (16, 17). The histone transfer mediated by DNA looping has also been suggested by nucleosome transcription studies with a prokaryotic RNAP (7, 8). Interestingly, the structures of the RNAP–nucleosome complexes containing the template DNA loop have been reported for mammalian and bacterial RNAPs (19, 20).

In the present study, we performed nucleosome transcription reactions *in vitro* and determined the cryo-EM structures of the RNAPII–nucleosome complexes, in which the upstream DNA region after RNAPII passage binds to the nucleosomal histones located in front of RNAPII. In these RNAPII–nucleosome complex structures, the upstream DNA is rewrapped in different forms from the previously reported ones. Interestingly, the DNA loop is not detected in the presence of the transcription elongation factors Spt4/5 and Elf1, which may sterically inhibit DNA rewrapping. These structures provide important information for understanding the biological relevance of the DNA loop formed when RNAPII is stalled on the DNA in the nucleosome.

## Results

### Cryo-EM structures of RNAPII–nucleosome complexes with upstream DNA looping

**Figure 1:**
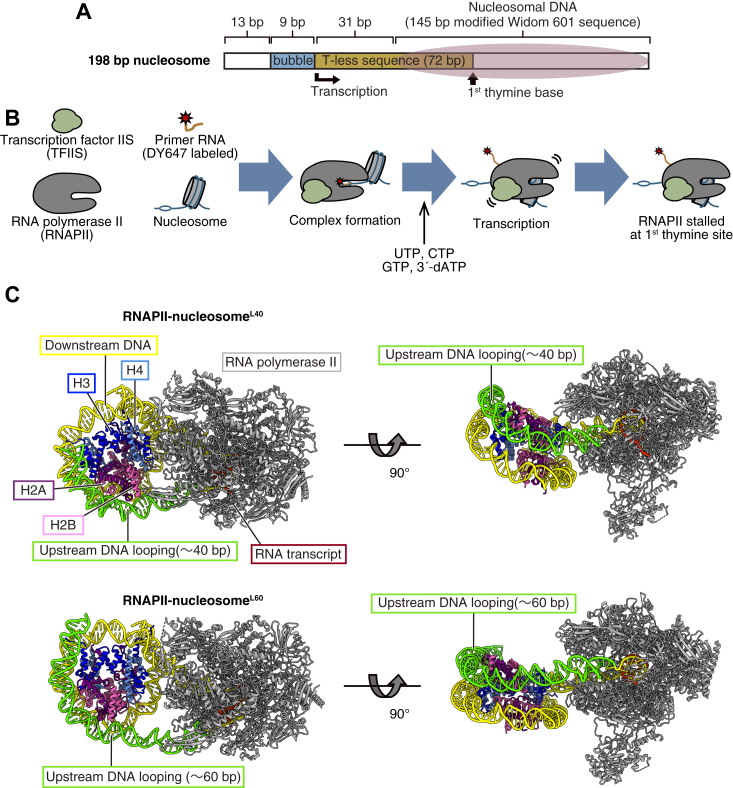
**Overall structures of RNAPII-nucleosome**^**L40**^**and RNAPII-nucleosome**^**L60**^. *A*, design of the nucleosome DNA template. *B*, scheme of the transcription reaction for the cryo-EM sample preparation. *C*, overall structures of RNAPII-nucleosome^L40^ and RNAPII-nucleosome^L60^. The figures show PDB models displayed as ribbons. Histones H2A, H2B, H3, and H4 are colored *purple*, *pink*, *blue*, and *light blue*, respectively. Upstream and downstream DNAs are colored *green* and *yellow*, respectively. RNAPII is colored *gray*. RNAPII, RNA polymerase II.

The nucleosome transcription reaction was conducted with purified *Komagataella pastoris* RNAPII in the presence of TFIIS, a transcription elongation factor that reactivates arrested or backtracked RNAPII. The nucleosome contained a 9 base-pair mismatched DNA region at the linker DNA to serve as a priming site for the RNA elongation reaction. We designed the nucleosomal DNA template in which RNAPII stalls when its catalytic center reaches the position 42 base-pairs from the nucleosome entry site, by incorporating 3′-dATP (Fig. 1, *A* and *B*). The nucleosome reconstitution (Fig. S1, *A* and *B*) and the transcription assay were conducted as described previously (4, 5), but the nucleosome: RNAPII ratio (2:1–1:1) and the NaCl concentration (0 mM to 100 mM) were changed (Fig. S1, *C* and *D*). The RNAPII–nucleosome complexes were then prepared by ultracentrifugation sedimentation with a sucrose and glutaraldehyde gradient (GraFix) for cryo-EM analysis (21) (Fig. S2, *A* and *B*).

We performed 3D reconstruction for the RNAPII–nucleosome complexes, in which RNAPII has proceeded 41 base pairs from the nucleosome entry site and is paused at the nucleosomal SHL(-1) position, with about 60 DNA base-pairs peeled from the histone surface (Figs. S2*C*, S3–S6, and S14). We determined two novel structures in which the transcribed upstream DNA behind RNAPII binds to the DNA-peeled surface of the nucleosome (Fig. 1*C*). We named these structures RNAPII-nucleosome^L40^ and RNAPII-nucleosome^L60^, which contain DNA loops with 41 and 62 base pairs of upstream DNA, respectively. The DNA loop formation may be enhanced in the presence of 100 mM NaCl, because it was not obvious under conditions without additional NaCl (4).

### Comparison of the RNAPII-nucleosome^L40^ and RNAPII-nucleosome^L60^ structures

**Figure 2:**
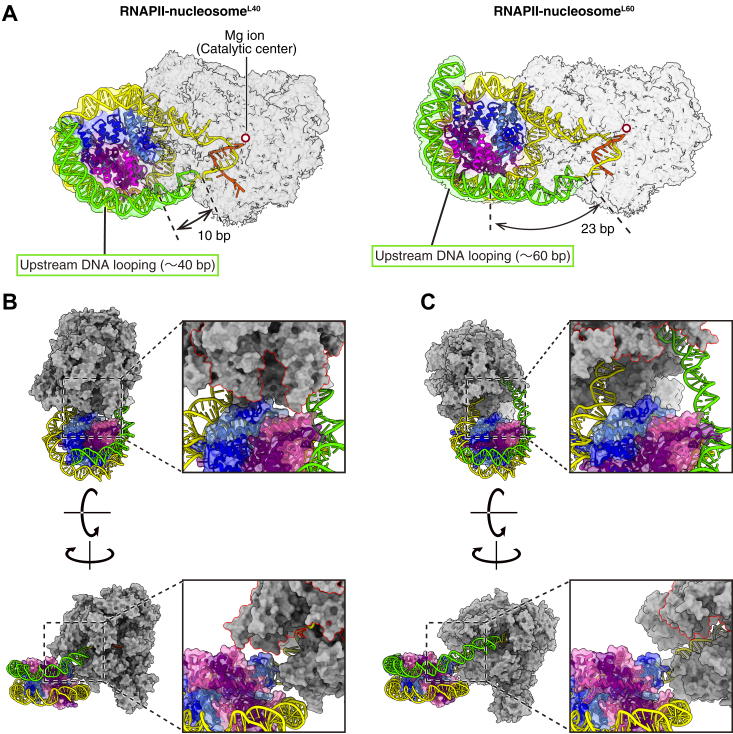
**Structural comparison of RNAPII-nucleosome**^**L40**^**and RNAPII-nucleosome**^**L60**^. *A*, details of the DNA paths of the RNAPII-nucleosome^L40^ and the RNAPII-nucleosome^L60^. The DNA and nucleosomes are shown as *ribbon models*, and the magnesium ions in the catalytic center are highlighted with *red circles* on the transparent composite maps. The colors of histones, DNA, and RNAPII are the same as in Figure 1. *B* and *C*, interactions between RNAPII and nucleosome in the RNAPII-nucleosome^L40^ (*B*) and RNAPII-nucleosome^L60^ (*C*) complexes. Two views are presented with close-ups (*right panels*). The colors of histones, DNA, and RNAPII are the same as in Figure 1. In the *lower right pane*l, the upstream DNA (*green*) is omitted to render the RNAPII-histone interface visible. RNAPII, RNA polymerase II.

In both structures, the upstream DNA is sharply kinked at the DNA exit site of RNAPII, and is folded back toward the downstream nucleosome (Fig. 2*A*). The upstream DNA spacers between RNAPII and the nucleosome are 10 base pairs and 23 base pairs in the RNAPII-nucleosome^L40^ and RNAPII-nucleosome^L60^ structures, respectively (Fig. 2*A*). In the RNAPII-nucleosome^L40^ structure, the nucleosomal histones are located close to RNAPII and may directly contact it (Fig. 2*B*). In contrast, the nucleosomal histones in the RNAPII-nucleosome^L60^ do not directly contact RNAPII (Fig. 2*C*). Therefore, the nucleosome of the RNAPII–nucleosome^L60^ complex may be more flexible than that of the RNAPII–nucleosome^L40^ complex. This structural difference between the RNAPII–nucleosome^L40^ and RNAPII–nucleosome^L60^ complexes may affect subsequent histone removal by RNAPII progression.

### Interactions of upstream DNA with nucleosomal histones

**Figure 3:**
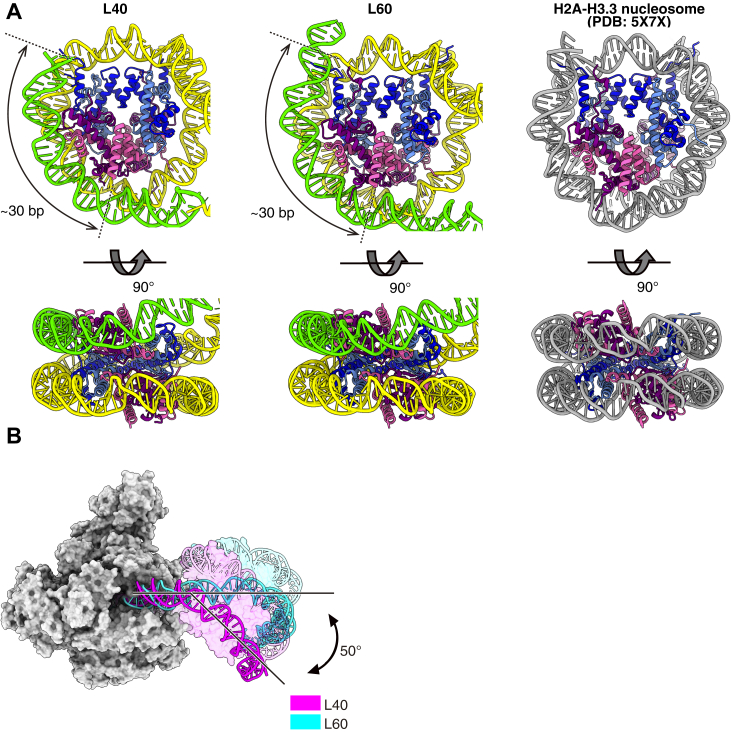
**The nucleosome structure with the upstream DNA loop**. *A*, structural comparison of the nucleosome region of the RNAPII-nucleosome^L40^, RNAPII-nucleosome^L60^, and the crystal structure of the H2A-H3.3 nucleosome (PDB: 5X7X) (22). The colors of histones are the same as in Figure 1.*B*, structural comparison of the upstream DNA regions of RNAPII-nucleosome^L40^ (*magenta*) and RNAPII-nucleosome^L60^ (*cyan*). The upstream DNA of the RNAPII-nucleosome^L40^ is kinked by ∼50 degrees relative to that of the RNAPII-nucleosome^L60^. RNAPII, RNA polymerase II.

In both the RNAPII-nucleosome^L40^ and RNAPII-nucleosome^L60^ structures, RNAPII is paused at the SHL(-1) position, and about 60 base pairs of the downstream nucleosomal DNA is peeled away by the RNAPII progression. The DNA-binding surface of the proximal H2A, H2B, and H3, which formerly contacted the DNA, becomes accessible and captures the upstream DNA. The length of the upstream DNA that directly contacts the nucleosomal histones is approximately 30 base pairs in both structures (Fig. 3*A*). The upstream DNA spacer of the RNAPII–nucleosome^L60^ complex is 13 base pairs longer than that of the RNAPII–nucleosome^L40^ complex (Fig. 2*A*). Consequently, the nucleosome orientation relative to RNAPII differs by about 50° between the RNAPII-nucleosome^L40^ and RNAPII-nucleosome^L60^ structures (Fig. 3*B*). The DNA-binding path of the upstream DNA bound to the histones is similar to that of the canonical nucleosome in these novel structures (22) (Fig. 3*A*).

### Spt4/5 and Elf1 may inhibit upstream DNA rewrapping

**Figure 4:**
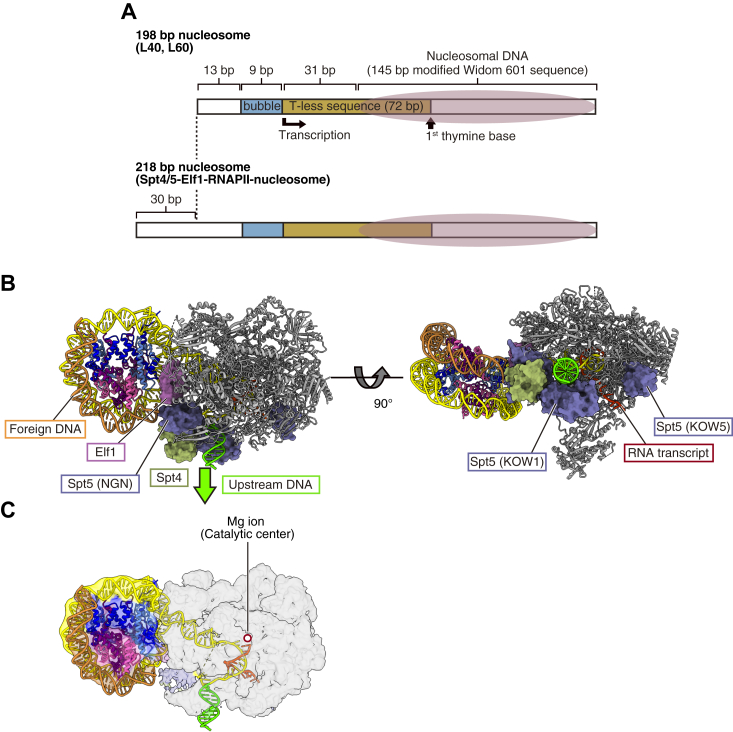
**Structure of Spt4/5-Elf1-RNAPII-nucleosome paused at the SHL(-1) position.***A*, designs of the 198 bp and 218 bp nucleosome DNA templates. *B*, cryo-EM structure of the Spt4/5-Elf1-RNAPII-nucleosome paused at the SHL(-1) position. The upstream DNA (*green*) is not kinked, as compared to the RNAPII-nucleosome^L40^ and RNAPII-nucleosome^L60^ structures. In addition, foreign DNA (*orange*) is observed near the DNA-peeled surface of the H2A-H2B dimer. *C*, details of the DNA path in the Spt4/5-Elf1-RNAPII-nucleosome paused at the SHL(-1) position. RNAPII, RNA polymerase II; SHL, superhelical location.

Spt4/5 and Elf1 (DSIF and ELOF1 for mammals, respectively) are constitutive factors for efficient transcription elongation (23, 24). We previously reported that Spt4/5 and Elf1 synergistically enhance the RNAPII processivity in nucleosome transcription (5). We next tested whether the upstream DNA loop structure could form in the presence of Spt4/5 and Elf1. To avoid a shortage of upstream DNA in the DNA loop formation, we extended the upstream DNA region by 30 base pairs, and conducted the nucleosome transcription in the presence of Spt4/5 and Elf1 (Figs. 4*A* and S1, *E*–*H*). We then determined the cryo-EM structure of the Spt4/5-Elf1-RNAPII–nucleosome complex paused at the SHL(-1) position (Figs. 4*B* and S7–S9). This structure is essentially the same as the previously reported Spt4/5-Elf1-RNAPII-nucleosome structure paused at the SHL(-1) position with the short upstream DNA template (5).

In this complex, Spt4/5 and Elf1 bind to the RNAPII surface (Fig. 4*B*). Interestingly, the Spt4 and Spt5 NGN domains are located near the DNA exit tunnel and interfere with the kinking of the upstream DNA, observed in the absence of Spt4/5 and Elf1 (Fig. 4*C*). Consequently, the upstream DNA orientation is directed away from the downstream nucleosome. Consistent with this, we could not find any structure containing an upstream DNA loop, although the binding of trans DNA (foreign DNA) to the exposed histone surface was observed (Fig. 4, *B* and *C*). In the mammalian RNAPII and DSIF, the DNA loop can reportedly be formed by the displacement of SPT4 and the NGN and KOW1 domains of SPT5 in the RNAPII–nucleosome complex, in the absence of the Elf1 homolog, ELOF1 (19). Elf1 may stabilize the Spt4/5 binding to RNAPII and contribute to the Spt4/5-mediated suppression of the upstream DNA looping. It is also possible that the formation of the DNA loop structure in the mammalian DSIF-RNAPII–nucleosome complex may be facilitated by the RNAPII progression up to the SHL(0) position, as reported previously (19).

To test whether the DNA looping is induced when RNAPII approaches the SHL(0) position, we designed a template nucleosome in which RNAPII pauses when it reaches SHL(0) (Figs. S1, *I* and *J* and S10, *A* and *B*). We performed nucleosome transcription by RNAPII in the presence of Spt4/5 and Elf1, and the RNAPII was intentionally paused when its catalytic center reached the position 54 base-pairs from the nucleosome entry site (Fig. S1, *K* and *L*). We then determined the cryo-EM structure of the Spt4/5-Elf1-RNAPII–nucleosome complex paused at the SHL(0) position (Figs. S10*C* and S11–S13). In this complex, the EM densities corresponding to Spt4, Spt5, and Elf1 are clearly observed, and the upstream DNA orientation is incompatible with DNA loop formation (Fig. S10*C*). Therefore, the DNA loop formation is unlikely to occur in the presence of Spt4/5 and Elf1, even when RNAPII is paused near the SHL(0) position of the nucleosome.

## Discussion

The present study has revealed the RNAPII-nucleosome structures rewrapping the upstream transcribed DNA, when the RNAPII is paused at the position 41 base-pairs from the nucleosome entry site. In this complex, about 60 base pairs of the nucleosomal DNA are peeled away, and the leading edge of RNAPII is located at the SHL(-1) position (Figs. 1, 2, and S14). A previous cryo-EM structure of the RNAPII–nucleosome complex, stalled at the 54 base-pair position, was reported with an upstream DNA loop (19). Surprisingly, our RNAPII-nucleosome^L40^ and RNAPII-nucleosome^L60^ structures are quite different from the previous structure paused at the 54 base pair position. In that structure, about 70 base pairs of the nucleosomal DNA are stripped, and the nucleosome is flipped by about 180°, as compared to our structures (Fig. S15). These facts suggest that the DNA looping can occur with a 10-bp periodicity at various SHLs, rather than a specific SHL. Two different (flipped/unflipped) types of DNA loops can be formed, probably depending on the RNAPII pausing position within the nucleosomal DNA.

In the present study, we could not obtain the RNAPII-nucleosome structure containing a flipped nucleosome configuration when the RNAPII was paused at the 54 base-pair position. This may be a consequence of the stable binding of Spt4/5 and Elf1 to RNAPII, as they restrict the upstream DNA orientation to prevent the DNA from rewrapping on the nucleosome (see below). The previously reported DNA loop structure with the flipped nucleosome configuration required the detachment of SPT4 and SPT5 from the RNAPII surface (19).

The DNA loop has been proposed to be an intermediate of histone transfer during transcription elongation (8, 10, 16, 17, 19). However, in the present study, we found that in the Spt4/5-Elf1-RNAPII-nucleosome structure, Spt4, the Spt5 NGN domain, and Elf1 bind near the DNA exit tunnel of RNAPII, and interfere with the DNA kinking. Consequently, the binding of Spt4/5 and Elf1 moves the upstream DNA away from the nucleosome and prevents the DNA loop formation. Alternatively, a previous report with mammalian DSIF (Spt4/5 homolog) and RNAPII showed that the DNA kinking at the DNA exit tunnel causes displacement of SPT4 and SPT5 from the RNAPII surface (19). These observations suggest that the DNA loop formation is incompatible with the binding of the elongation factors. Therefore, the DNA loop-mediated histone transfer may occur during the transcription elongation by RNAPII without elongation factors or concomitantly with the SPT4/5 detachment. Further studies are awaited to solve this issue.

We previously determined the snapshot cryo-EM structures of RNAPII–nucleosome complexes, in which Spt6, Spn1, and the Paf1 complex were bound to the RNAPII together with Spt4/5 and Elf1, and explained the mechanism by which the RNAPII elongation complex promotes nucleosome disassembly and reassembly with the aid of the histone chaperone FACT (18). In this series of structures, we did not find the complex with the DNA loop. Therefore, the DNA looping pathway described in the present and previous studies may be an alternative pathway to the nucleosome transfer by RNAPII with transcription elongation factors (18, 19). Histone chaperones, such as FACT, may be required in both pathways.

Previous low-resolution images obtained by conventional EM with a bacterial RNAP and nucleosome suggested that DNA damage in the nucleosome induces the DNA loop formation to stall RNAP on the damaged nucleosome (20). This finding implies that the DNA looping may function as a part of transcription-coupled DNA repair, in which RNAPII plays an essential role to detect DNA lesions in chromatin (25). In the future, it will be intriguing to study the functional consequences of DNA looping in the RNAPII–nucleosome complexes during the histone transfer in transcription elongation and/or DNA lesion recognition, in the course of transcription-coupled DNA repair processes.

## Experimental procedures

### Protein expression and preparation

Recombinant histones were prepared as described previously (26). In brief, pET-15b plasmids (Novagen) encoding human histones H2A, H2B, H3.3, and H4 were introduced into *Escherichia coli* strain BL21 (DE3) (for H2A, H2B and H3.3) or JM109 (DE3) (for H4). The histones were produced as N-terminally hexahistidine (His_6_)-tagged proteins and denatured in guanidine buffer [50 mM Tris-HCl (pH 8.0), 500 mM NaCl, 7 M guanidine-HCl, and 5% glycerol]. After denaturation, the histones were purified by Ni-NTA affinity chromatography (QIAGEN) with 50 mM Tris-HCl (pH 8.0) buffer, containing 500 mM NaCl, 6 M urea, 5% glycerol, and 5 mM to 500 mM imidazole. The histones were then dialyzed against 10 mM Tris-HCl (pH 8.0) buffer containing 2 mM β-mercaptoethanol. After the dialysis, the His_6_-tag was removed by thrombin protease (Wako). The resulting histone proteins were further purified by MonoS cation exchange chromatography (Cytiva) under denaturing conditions with 20 mM CH_3_COONa (pH 5.2) buffer, containing 200 mM to 900 mM NaCl, 6 M urea, 5 mM β-mercaptoethanol, and 1 mM EDTA. The purified histones were desalted by dialysis against a 2 mM β-mercaptoethanol solution, freeze-dried, and stored as powders at 4 °C.

*Komagataella pastris* RNAPII, TFIIS, Spt4/5, and Elf1 were purified as described previously (23, 27, 28). In brief, RNAPII containing the TAP-tagged Rpb2 subunit was purified by Q Sepharose Fast Flow column anion exchange chromatography (Cytiva), anti-FLAG affinity chromatography, and Resource Q column anion exchange chromatography (Cytiva). TFIIS, Spt4/5, and Elf1 were expressed as His_6_-tagged proteins in *E*. *coli* strain KRX (Promega) and purified by COSMOGEL His-Accept column (Nacalai Tesque) affinity chromatography. The His_6_-tag was then removed using HRV-3C protease. After the tag removal, these proteins were further purified by Resource S (Cytiva) cation exchange chromatography, concentrated, and stored at −80 °C.

### Preparation of the DNA fragment for nucleosome reconstitution

The modified Widom 601 sequence DNA fragment for the RNAPII–nucleosome and Spt4/5-Elf1-RNAPII–nucleosome complexes, SHL(-1) stop, was prepared as described previously (4, 29). Briefly, the SHL(-1) stop fragment was cloned into the pGEM-T Easy vector and amplified in the *E. coli* strain DH5α. The DNA fragment was extracted and purified by polyethylene glycol precipitation and DEAE anion exchange chromatography (TOSOH). The sequences of the DNA fragments are as follows:

Nontemplate strand:

5′- TGGCCGTTTTCGTTGTTTTTTTCTGTCTCGTGCCTGGTGTCTTGGGTGTAATCCCCTTGGCGGTTAAAACGCGGGGGACAGCGCGTACGTGCGTTTAAGCGGTGCTAGAGCTGTCTACGACCAATTGAGCGGCCTCGGCACCGGGATTCTGAT -3′

Template strand:

5′- ATCAGAATCCCGGTGCCGAGGCCGCTCAATTGGTCGTAGACAGCTCTAGCACCGCTTAAACGCACGTACGCGCTGTCCCCCGCGTTTTAACCGCCAAGGGGATTACACCCAAGACACCAGGCACGAGACAGAAAAAAACAACGAAAACGGCCACCA -3′.

The modified Widom 601 sequence for the Spt4/5-Elf1-RNAPII–nucleosome complex (SHL(0) stop) was prepared as described previously (18). The DNA fragment containing the required sequence was amplified and cleaved with *Bgl*I (Takara). After the cleavage, the resulting DNA fragment was purified by non-denaturing PAGE using a Prep Cell apparatus (Bio-Rad). The purified DNA fragment was concentrated by Amicon Ultra 3K centrifugal filters (Millipore) and stored at −20 °C. The sequences of the DNA fragment are as follows:

Nontemplate strand:

5′- TGGCCGTTTTCGTTGTTTTTTTCTGTCTCGTGCCTGGTGTCTTGGGTGTTTTCCCCTTGGCAAAAAAAACGCGGGGGACAGCGCGTACGTGCGTTTAAGCGGTGCTAGAGCTGTCTACGACCAATTGAGCGGCCTCGGCACCGGGATTCTGAT -3′

Template strand:

5′- ATCAGAATCCCGGTGCCGAGGCCGCTCAATTGGTCGTAGACAGCTCTAGCACCGCTTAAACGCACGTACGCGCTGTCCCCCGCGTTTTTTTTGCCAAGGGGAAAACACCCAAGACACCAGGCACGAGACAGAAAAAAACAACGAAAACGGCCA -3′.

### Nucleosome reconstitution and purification for *in vitro* transcription

The histone octamer was reconstituted as described previously (26). Histones H2A, H2B, H3.3, and H4 were mixed in a 1:1:1:1 molar stoichiometry in guanidine-containing denaturing buffer [20 mM Tris-HCl (pH 7.5), 7 M guanidine-HCl, and 20 mM β-mercaptoethanol], and dialyzed against refolding buffer (10 mM Tris-HCl (pH 7.5), 2 M NaCl, 1 mM EDTA, and 5 mM β-mercaptoethanol). After the dialysis, the histone octamer was purified by HiLoad Superdex 200 pg gel filtration chromatography (Cytiva).

The nucleosome was reconstituted with the histone octamer and the DNA fragment by the salt-dialysis method (26). The 42 and 72 bp bubble DNA fragments containing the 9 base-pair mismatched region were ligated to the cohesive end of the nucleosomal DNA by T4 DNA ligase (NIPPON GENE), as described previously (4). The sequences of the bubble DNA fragments are as follows:

42 bp bubble DNA fragment nontemplate strand:

5′- GCTTACGTCAGTCTGGCCATCTTTGTGTTTGGTGTGTTTGGGTGG -3′

42 bp bubble DNA fragment template strand:

5′- CCCAAACACACCAAACACAAGAGCTAATTGACTGACGTAAGC -3′

72 bp bubble DNA fragment nontemplate strand:

5′- CCTCTGCCTTTAAAGCAATAGGAGGTCCACGCTTACGTCAGTCTGGCCATCTTTGTGTTTGGTGTGTTTGGGTGG -3′

72 bp bubble DNA fragment template strand:

5′- CCCAAACACACCAAACACAAGAGCTAATTGACTGACGTAAGCGTGGACCTCCTATTGCTTTAAAGGCAGAGG -3′.

The nucleosome with a linker DNA containing a bubble region was purified by preparative non-denaturing PAGE, using a Prep Cell apparatus (Bio-Rad). The purified nucleosome was concentrated using Amicon Ultra 30K centrifugal filters (Millipore) and stored at −80 °C.

### Preparation of RNAPII–nucleosome complexes for cryo-EM analyses

The RNAPII–nucleosome and Spt4/5-Elf1-RNAPII–nucleosome complexes for cryo-EM analyses were prepared as described previously, with some modifications (4, 5). The RNAPII–nucleosome complex transcription reaction was conducted by mixing 0.1 μM nucleosome, 0.1 μM RNAPII, 0.1 μM TFIIS, and 0.4 μM primer RNA (5′-DY647- AUAAUUAGCUC-3′) (Dharmacon) in the reaction mixture [26 mM HEPES-KOH (pH 7.5), 50 mM CH_3_COOK, 5 mM MgCl_2_, 0.2 μM (CH_3_COO)_2_Zn, 0.4 mM UTP, 0.4 mM CTP, 0.4 mM GTP, 5 μM 3′-dATP, 1.5% glycerol, 0.02 μM TCEP-HCl, and 0.1 mM DTT], for 30 min at 30 °C. The Spt4/5-Elf1-RNAPII–nucleosome complexes were prepared by adding 0.4 μM Spt4/5 and 1.0 μM Elf1 to this preparation in the reaction mixture [28 mM HEPES-KOH (pH 7.5), 50 mM CH_3_COOK, 5 mM MgCl_2_, 0.3 μM (CH_3_COO)_2_Zn, 0.4 mM UTP, 0.4 mM CTP, 0.4 mM GTP, 5 μM 3′-dATP, 2% glycerol, 0.03 μM TCEP-HCl, and 0.1 mM DTT]. After the transcription reaction, 100 mM NaCl was added to the reaction mixture, which was further incubated for 30 min at 30 °C. The reaction mixture was then fractionated by ultracentrifugation sedimentation on a sucrose and glutaraldehyde gradient (GraFix) (21). The sucrose and glutaraldehyde gradient [20 mM HEPES-KOH (pH 7.5), 50 mM CH_3_COOK, 0.2 μM (CH_3_COO)_2_Zn, 0.1 mM TCEP-HCl, 10% to 20% sucrose, and 0% to 0.1% glutaraldehyde] was prepared using an SG gradient maker (Cytiva) and a peristaltic pump. The transcription reaction mixture was applied on the top of the gradient solution and ultracentrifuged at 27,000 rpm at 4 °C for 16 h, using an SW41 rotor (Beckman Coulter). The fractionated samples containing the RNAPII–nucleosome complexes were desalted using a PD-10 column (Cytiva) in 20 mM HEPES-KOH (pH 7.5) buffer, containing 50 mM CH_3_COOK, 0.2 μM (CH_3_COO)_2_Zn, and 0.1 mM TCEP-HCl. The resulting sample was concentrated with Amicon Ultra 100K centrifugal filters (Millipore). The final dsDNA concentrations of the complex were 91.4 ng/μl for RNAPII-nucleosome^L40^ and RNAPII-nucleosome^L60^, 117 ng/μl for Spt4/5-Elf1-RNAPII-nucleosome (SHL(-1) stop), and 50 ng/μl for Spt4/5-Elf1-RNAPII-nucleosome (SHL(0) stop). The qualities of the sample preparations are shown in Fig. S1. The RNAPII–nucleosome^L40^, RNAPII–nucleosome^L60^, and Spt4/5-Elf1-RNAPII–nucleosome complex (SHL(-1) stop) preparations were supplemented with 0.005% Tween-20 to improve the particle orientation distribution. These complexes were then applied to Quantifoil R1.2/1.3, Cu, 200 mesh grids (Quantifoil Micro Tools GmbH), which were glow-discharged using a PIB-10 ION Bombarder (Vacuum Device). The grid was plunge-frozen using a Vitrobot Mark IV (Thermo Fisher Scientific) at 4 °C and 100% humidity.

### Cryo-EM data collection and image processing

Cryo-EM images were recorded by a Krios G4 transmission electron microscope (Thermo Fisher Scientific), equipped with a K3 direct electron detector and a BioQuantum energy filter (Gatan) with a slit width of 20 eV. Details of the processing statistics are provided in Table S1. In total, 9189 movies for RNAPII-nucleosome^L40^ and RNAPII-nucleosome^L60^, 11,047 movies for Spt4/5-Elf1-RNAPII-nucleosome (SHL(-1) stop), and 17,348 movies for Spt4/5-Elf1-RNAPII-nucleosome (SHL(0) stop) were acquired using the EPU automation software (Thermo Fisher Scientific). For data acquisition, the pixel size was 1.06 Å, the defocus values were between −1.0 and −2.5 μm, and the dose was 1.496 e^-^/Å^2^ for RNAPII-nucleosome^L40^ and RNAPII-nucleosome^L60^, 1.512 e^-^/Å^2^ for Spt4/5-Elf1-RNAPII-nucleosome (SHL(-1) stop), and 1.567 e^-^/Å^2^ for Spt4/5-Elf1-RNAPII-nucleosome (SHL(0) stop) per frame over 40 frames. Motion correction was conducted using MotionCor2 (30), and CTF estimation was conducted using CTFFIND4 (31). For the Spt4/5-Elf1-RNAPII–nucleosome complex (SHL(-1) stop) dataset, the movie frames were aligned before summing into the integrated images on DigitalMicrograph (Gatan). All subsequent image processing was performed by RELION 3.1 (32) and RELION 4.0 (33). Schemes are shown in Fig. S2*C* for RNAPII-nucleosome^L40^ and RNAPII-nucleosome^L60^, Fig. S7*C* for Spt4/5-Elf1-RNAPII–nucleosome complex (SHL(-1) stop), and Fig. S11*C* for Spt4/5-Elf1-RNAPII-nucleosome (SHL(0) stop).

The RNAPII-nucleosome^L40^ and RNAPII-nucleosome^L60^ particles were picked using RELION auto-picking based on a Laplacian of Gaussian filter and extracted with a binning factor of 2 (pixel size of 2.12 Å/pixel). These particles were subjected to 2D classification, RNAPII–nucleosome complex class selection, and 3D classification using a low-pass filtered map (EMDB-9713) as the reference model (5). The RNAPII–nucleosome complex class that forms the DNA loop was selected as a reference for Topaz picking (34). Particles were repicked using Topaz and subjected to 2D classification to remove junk particles. After the 3D classification, 51,652 and 97,768 particles were selected as the RNAPII-nucleosome^L40^ and the RNAPII-nucleosome^L60^, respectively. Each class was re-extracted with a binning factor of 1 (pixel size of 1.06 Å/pixel). The RNAPII-nucleosome^L40^ and RNAPII-nucleosome^L60^ particles were subjected to 3D refinement, followed by CTF refinement and Bayesian polishing. Focused refinements of both RNAPII and nucleosome were separately performed, using specific masks. These focused-refined maps were combined using phenix.combine_focused_maps in the Phenix software package (35).

For the Spt4/5-Elf1-RNAPII-nucleosome complex (SHL(-1) stop), the image processing to 3D classification was the same as described above. The selected classes from 3D classification were re-extracted with a binning factor of 1 (pixel size of 1.06 Å/pixel). Particles were subtracted with a mask for Spt4/5, followed by 3D classification. The selected classes containing Spt4/5 were reverted, refined, and resubtracted with a nucleosome mask. After the 3D classification with the nucleosome, 74,079 particles were selected as the Spt4/5-Elf1-RNAPII-nucleosome. The map of the nucleosome was refined and postprocessed. Subtracted particles were reverted, followed by refinement and postprocessing for the overall structure. The subtraction with an RNAPII mask was applied to the overall structure and then refined and postprocessed as the RNAPII structure. This focused-refined map was combined using phenix.combine_focused_maps in the Phenix software package (35).

For the Spt4/5-Elf1-RNAPII-nucleosome complex (SHL(0) stop), particles were picked using RELION auto-picking based on a Laplacian of Gaussian filter, and extracted with a binning factor of 4 (pixel size of 4.24 Å/pixel). These particles were subjected to 2D classification, RNAPII-nucleosome complex class selection, and 3D classification using a low-pass filtered map (EMDB-6747) as the reference model (23). After the 3D classification, selected particles were subtracted with a mask for the whole RNAPII-Spt4/5-Elf1 complex. Subtracted particles were re-extracted with a binning factor of 2 (pixel size of 2.12 Å/pixel), refined, and classified as without alignment 3D classification with a mask covering both Spt4/5 and Elf1. The selected classes containing Spt4/5 and Elf1 were reverted, refined, and classified as with alignment 3D classification. The Spt4/5 and Elf1 binding classes were selected and resubtracted with a nucleosome mask made from another class with well-observed nucleosome density. Resubtracted classes from 3D classification were re-extracted with a binning factor of 1 (pixel size of 1.06 Å/pixel), refined, and classified with alignment 3D classification. The class that contained nucleosome density (12,176 particles) was selected as the Spt4/5-Elf1-RNAPII–nucleosome complex (SHL(0) stop), and these particles were subjected to 3D refinement.

The final resolutions of the refined 3D maps of the RNAPII-nucleosome^L40^, the RNAPII-nucleosome^L60^, the Spt4/5-Elf1-RNAPII-nucleosome (SHL(-1) stop), and the Spt4/5-Elf1-RNAPII-nucleosome (SHL(0) stop) were 3.7 Å, 3.2 Å, 5.7 Å, and 6.7 Å, respectively, as estimated by the gold standard Fourier shell correlation (FSC) at an FSC = 0.143 (36). The 3D sphericity values were calculated on the 3DFSC server (37). The Map-To-Model FSC curve was calculated using Phenix (35). The local resolutions of the RNAPII-nucleosome^L40^, the RNAPII-nucleosome^L60^, the Spt4/5-Elf1-RNAPII-nucleosome complex (SHL(-1) stop), and the Spt4/5-Elf1-RNAPII-nucleosome (SHL(0) stop) were calculated by RELION 4.0.

### Model building

The overall atomic models of the RNAPII-nucleosome^L40^ and RNAPII-nucleosome^L60^ were built based on the cryo-EM structure of the RNAPII-nucleosome complex (PDB ID: 6A5T) (4), and the overall atomic model of the Spt4/5-Elf1-RNAPII–nucleosome complex (SHL(-1) stop) was built based on the cryo-EM structure of the Spt4/5-Elf1-RNAPII-nucleosome complex (PDB ID: 6J4X) (5). The models of amino acids, DNA, and RNA were modified using COOT (38) and ISOLDE (39). The final structure was evaluated with the MolProbity (40) program (Table S1). Q-scores (41) were obtained from wwPDB validation and are shown in Table S1. All structural figures were prepared with UCSF Chimera (42) and ChimeraX (43).

## Data availability

The cryo-EM maps and coordinates have been deposited in the Electron Microscopy Data Bank (EMDB) and Protein Data Bank (PDB), respectively, with accession codes: EMD-36252 and 8JH3 (RNAPII-nucleosome^L40^), EMD-36253 and 8JH4 (RNAPII-nucleosome^L60^), EMD-36251 and 8JH2 (Spt4/5-Elf1-RNAPII-nucleosome (SHL(-1) stop)), and EMD-37848 (Spt4/5-Elf1-RNAPII-nucleosome (SHL(0) stop)).

## Supporting information

This article contains supporting information.

## Conflict of interest

The authors declare that they have no conflicts of interests with the contents of this article.
