# Supplementary material for: Cryo-EM structures of RNA polymerase II–nucleosome complexes rewrapping transcribed DNA
Source: J Biol Chem. 2023 Nov 17;299(12):105477. doi: 10.1016/j.jbc.2023.105477 (PMC10703601; doi:10.1016/j.jbc.2023.105477)
Supplement: Supplemental Figure and Table [file mmc1.pdf]

Supporting information

Cryo-EM structures of RNA polymerase II-nucleosome complexes rewrapping transcribed DNA

Munetaka Akatsu[1,2,4], Haruhiko Ehara[3,4], Tomoya Kujirai[1,3], Risa Fujita[1,5], Tomoko Ito[1],Ken Osumi[1], Mitsuo Ogasawara[1], Yoshimasa Takizawa[1], Shun-ichi Sekine[3*], and Hitoshi Kurumizaka[1,2,3*]

1 Laboratory of Chromatin Structure and Function, Institute for Quantitative Biosciences, The University of Tokyo, 1-1-1 Yayoi, Bunkyo-ku, Tokyo 113-0032, Japan.

2 Department of Biological Sciences, Graduate School of Science, The University of Tokyo, 1-1-1 Yayoi, Bunkyo-ku, Tokyo 113-0032, Japan.

3 Laboratory for Transcription Structural Biology, RIKEN Center for Biosystems Dynamics Research, 1-7-22 Suehiro-cho, Tsurumi-ku, Yokohama 230-0045, Japan.

4 These authors contributed equally to this work.

5 Present address: Research Organization for Nano & Life Innovation, Waseda University, 513 Waseda-tsurumaki-cho, Shinjuku-ku, Tokyo 162-0041, Japan.

*To whom correspondence should be addressed. Tel: +81-45-503-9204; Fax: +81-45-503-9201; Email: shunichi.sekine@riken.jp or Tel: +81-3-5841-7826; Fax: +81-3-5847-1468; Email: kurumizaka@iqb.u-tokyo.ac.jp

This PDF file includes: Figure S1-15, Table S1

Figure S1

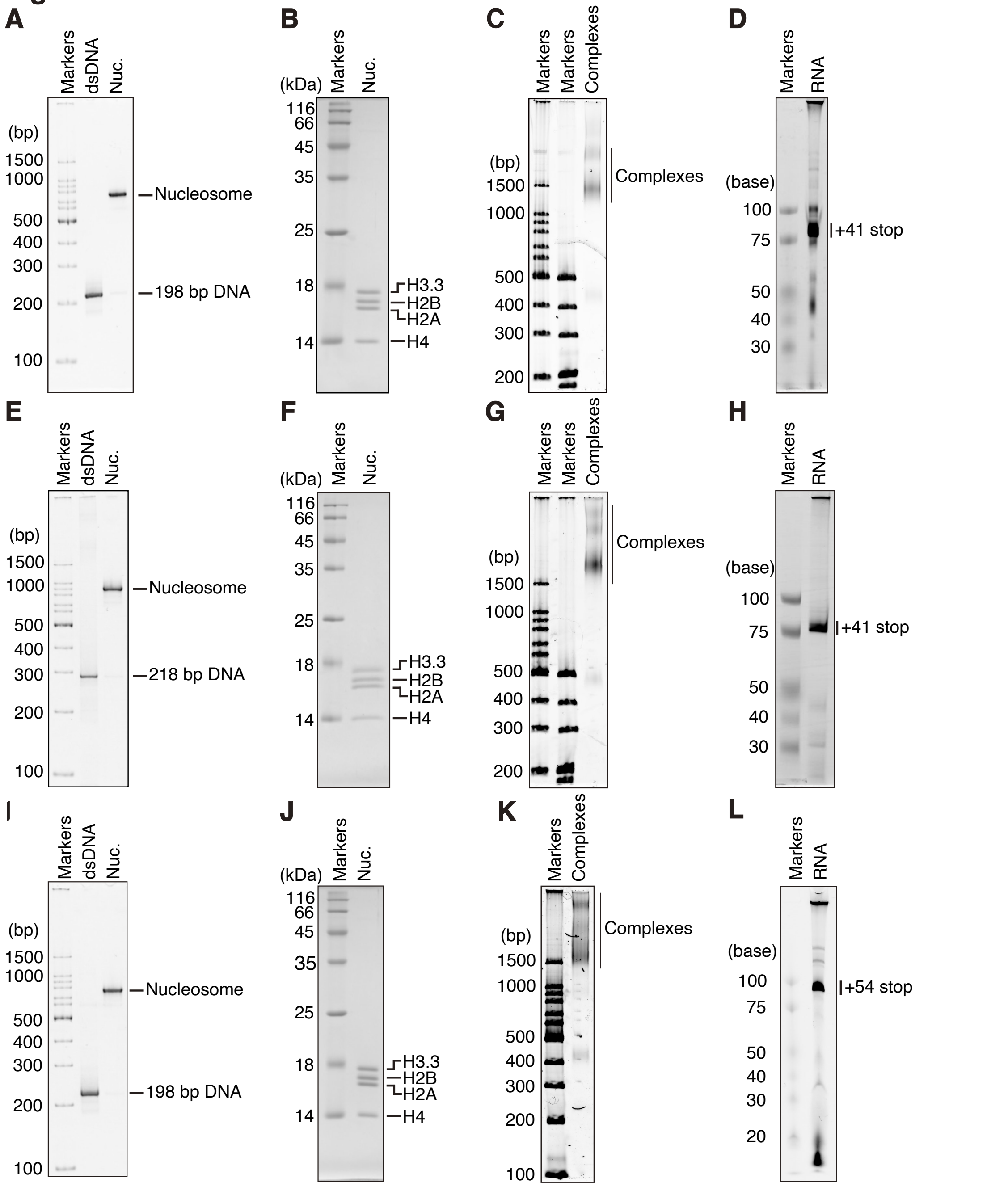

Figure S1: Sample qualities of nucleosomes, RNAPII-nucleosome complexes, and RNA transcripts from RNAPII-nucleosome complexes

(A) Non-denaturing PAGE analysis of purified 198 bp (+42 stop) nucleosome. Bands are stained by ethidium bromide.
(B) SDS-PAGE analysis of purified 198 bp (+42 stop) nucleosome. Bands are stained Coomassie Brilliant Blue.
(C) Non-denaturing PAGE analysis of RNAPII-nucleosome complexes before the grid preparation. Bands are stained by SYBR Gold.
(D) Denaturing PAGE analysis of RNA transcripts from RNAPII-nucleosome complexes. Bands are detected by DY647 fluorescent dye.
(E) Non-denaturing PAGE analysis of purified 218 bp (+42 stop) nucleosome. Bands are stained by ethidium bromide.
(F) SDS-PAGE analysis of purified 218 bp (+42 stop) nucleosome. Bands are stained Coomassie Brilliant Blue.
(G) Non-denaturing PAGE analysis of Spt4/5-Elf1-RNAPII-nucleosome (SHL(-1) stop) complexes before the grid preparation. Bands are stained by SYBR Gold.
(H) Denaturing PAGE analysis of RNA transcripts from Spt4/5-Elf1-RNAPII-nucleosome (SHL(-1) stop) complexes. Bands are detected by DY647 fluorescent dye.
(I) Non-denaturing PAGE analysis of purified 198 bp (+54 stop) nucleosome. Bands are stained by ethidium bromide.
(J) SDS-PAGE analysis of purified 198 bp (+54 stop) nucleosome. Bands are stained Coomassie Brilliant Blue.
(K) Non-denaturing PAGE analysis of Spt4/5-Elf1-RNAPII-nucleosome (SHL(0) stop) complexes before the grid preparation. Bands are stained by SYBR Gold.
(L) Denaturing PAGE analysis of RNA transcripts from (SHL(0) stop) complexes. Bands are detected by DY647 fluorescent dye.

Figure S2

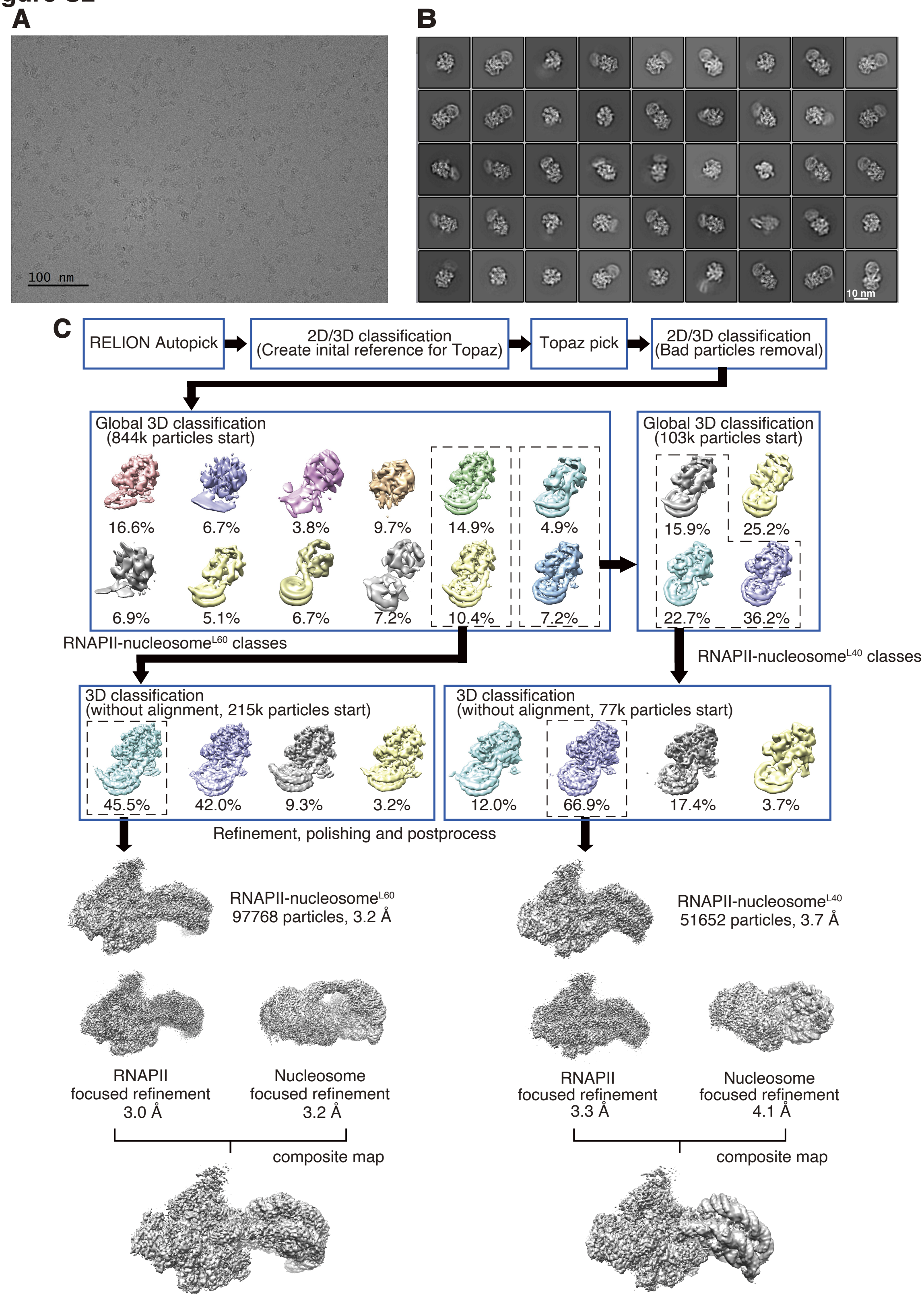

Figure S2: Cryo-EM analysis of RNAPII-nucleosome^{L40} and RNAPII-nucleosome^{L60}.
(A) Representative micrograph of the RNAPII-nucleosome^{L40} and RNAPII-nucleosome^{L60} datasets.
(B) Representative 2D class averages from the 2D classification after the Topaz picking.
(C) Image processing scheme of the RNAPII-nucleosome^{L40} and RNAPII-nucleosome^{L60} structures.

Figure S3

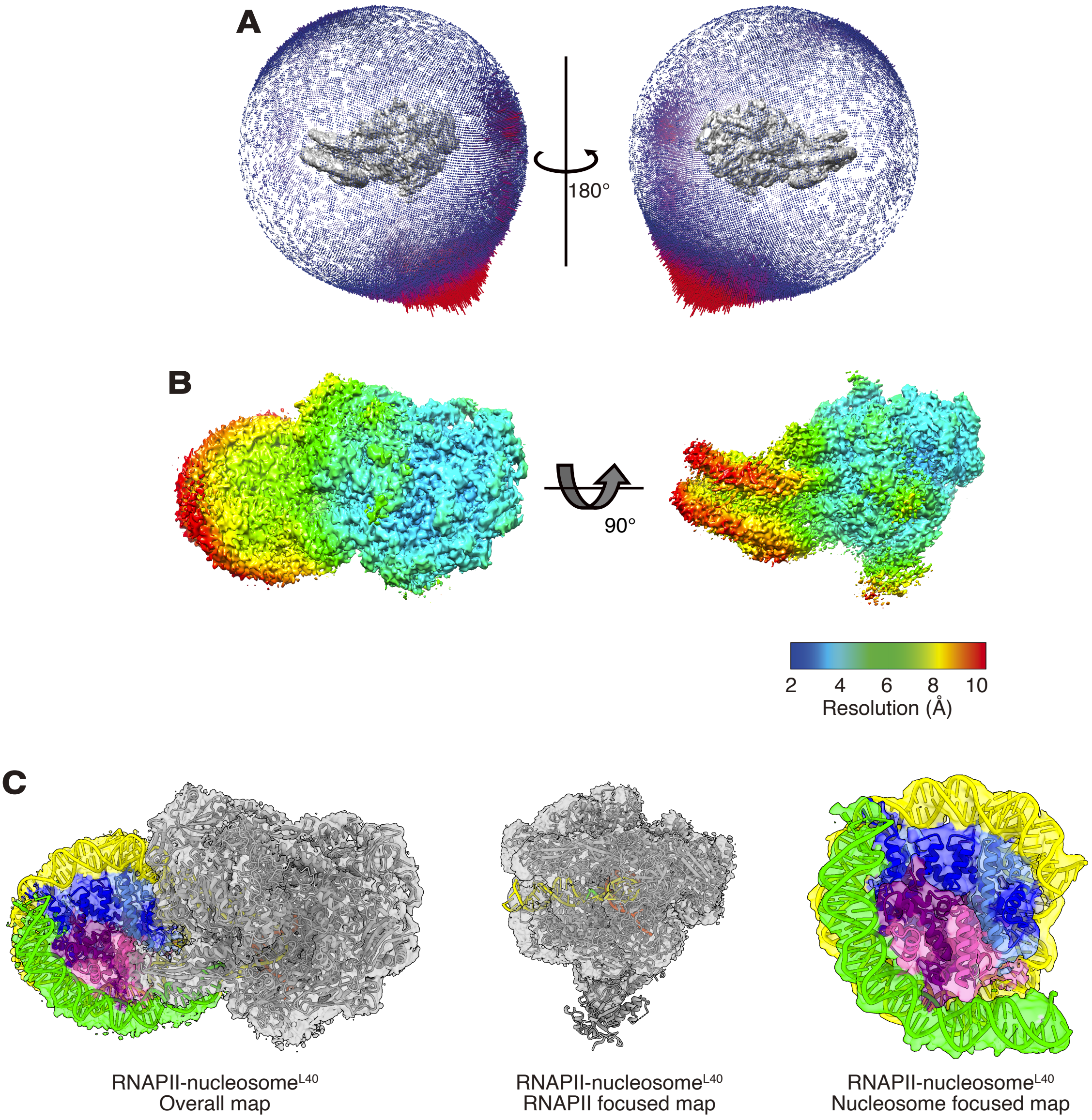

Figure S3: Cryo-EM map qualities of RNAPII-nucleosome^{L40}.
(A) Angular distributions of RNAPII-nucleosome^{L40}.
(B) Local resolution maps of RNAPII-nucleosome^{L40}.
(C) Model-to-density fits of the RNAPII-nucleosome^{L40}.

Figure S4

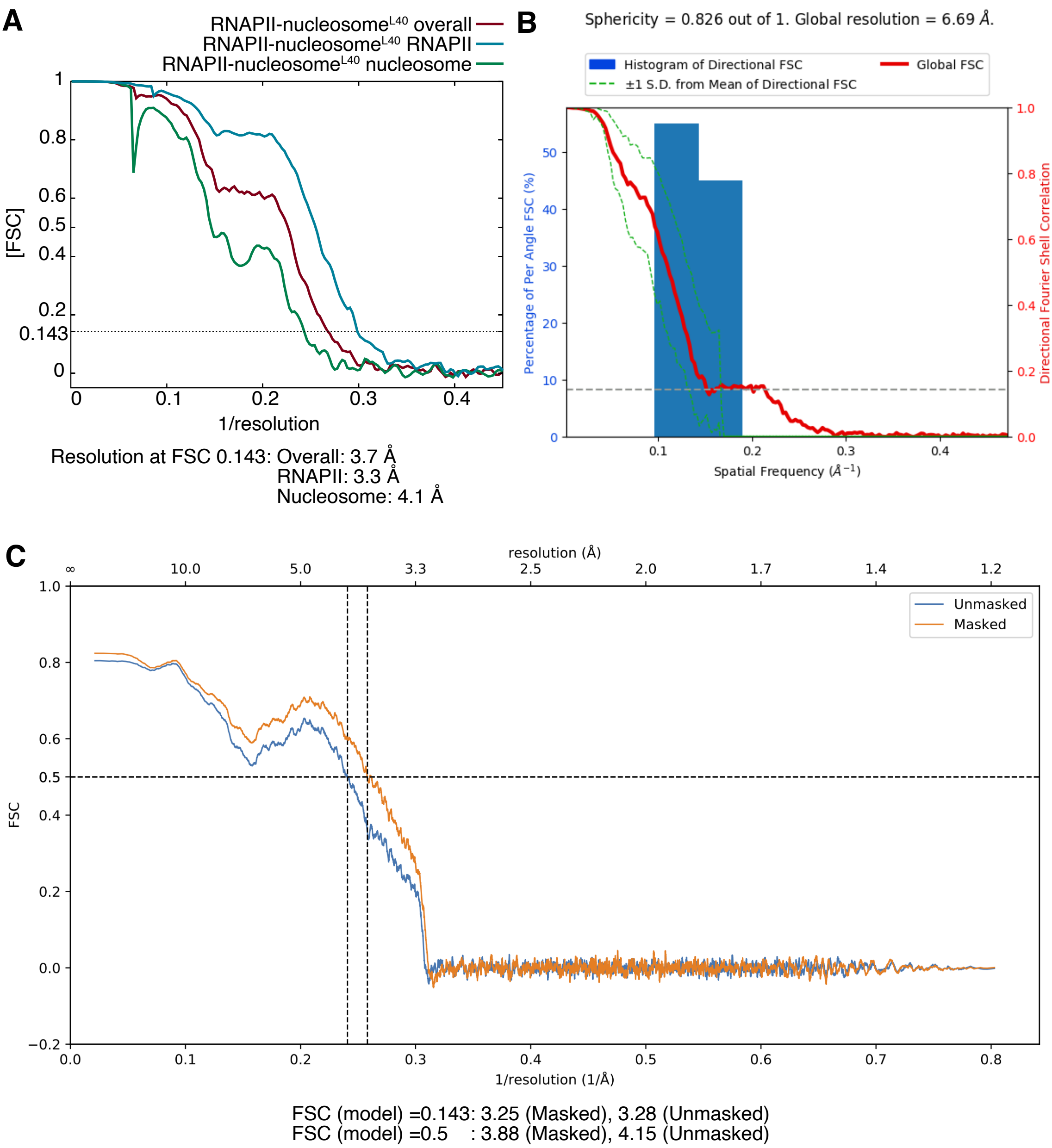

Figure S4: Cryo-EM data qualities of RNAPII-nucleosome^{L40}.
(A) Gold-standard Fourier Shell Correlations (FSCs) of RNAPII-nucleosome^{L40}. These curves were calculated by RELION post-processing.
(B) Histogram and Directional FSC plots of RNAPII-nucleosome^{L40}. These curves were calculated on the 3DFSC server.
(C) The Map-To-Model FSC curve of RNAPII-nucleosome^{L40}. These curves were calculated using Phenix.

Figure S5

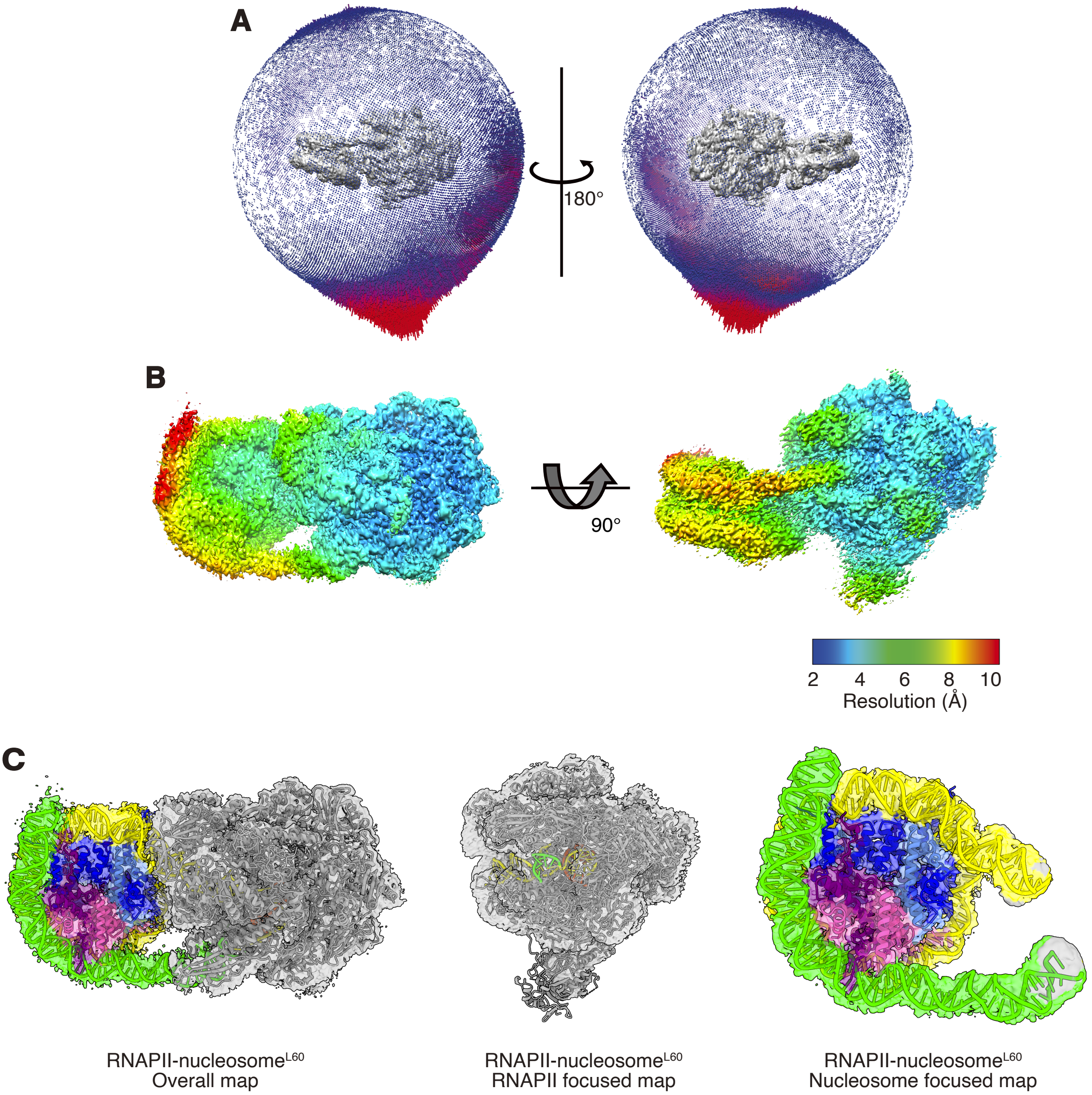

Figure S5: Cryo-EM map qualities of RNAPII-nucleosome^{L60}.
(A)Angular distributions of RNAPII-nucleosome^{L60}.
(B) Local resolution maps of RNAPII-nucleosome^{L60}.
(C) Model-to-density fits of the RNAPII-nucleosome^{L60}.

Figure S6

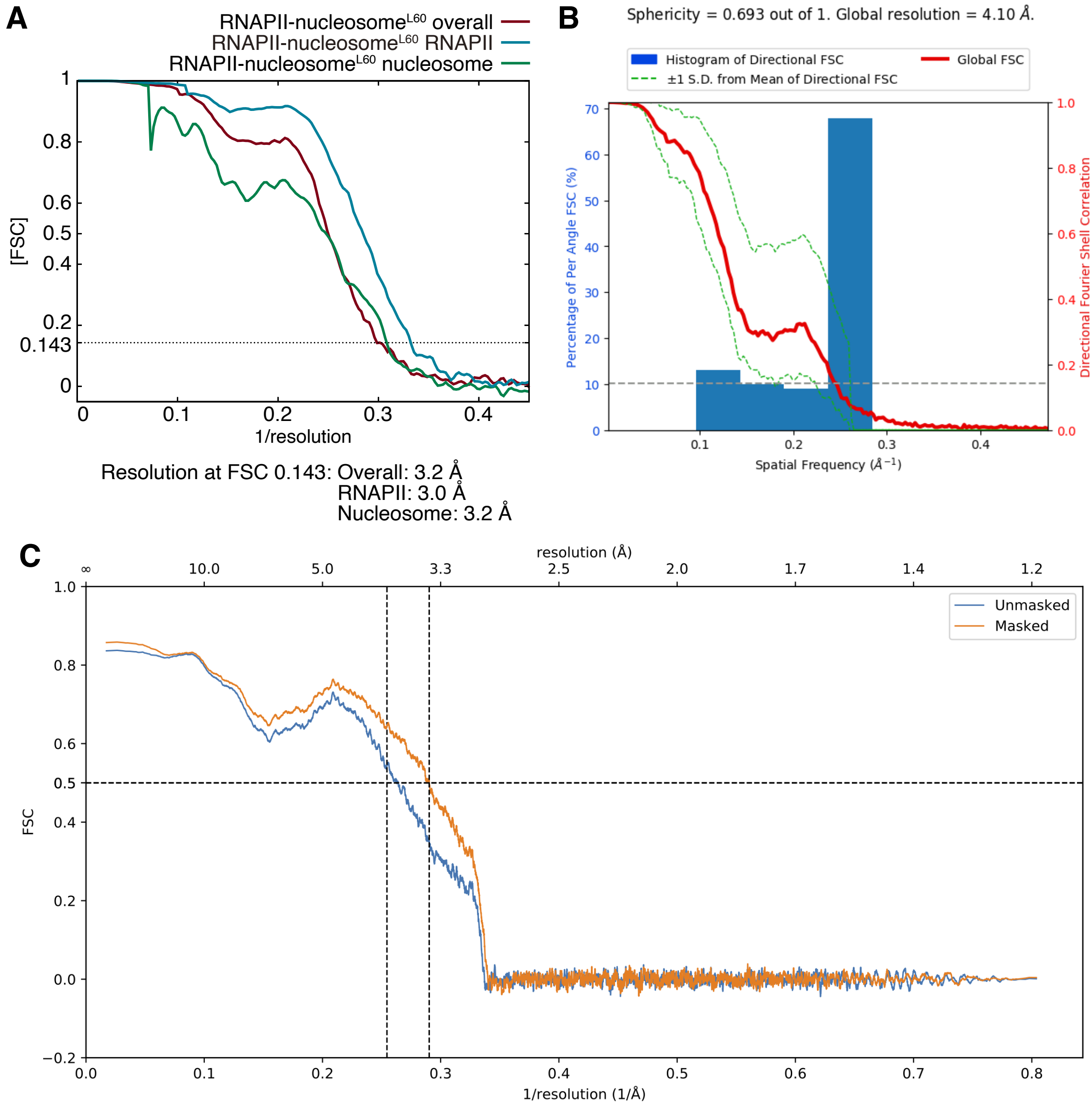

FSC (model) =0.143: 2.98 (Masked), 3.01 (Unmasked)
FSC (model) =0.5 : 3.44 (Masked), 3.93 (Unmasked)

Figure S6: Cryo-EM data qualities of RNAPII-nucleosome^{L60}.
(A) Gold-standard Fourier Shell Correlations (FSCs) of RNAPII-nucleosome^{L60}.
These curves were calculated by RELION post-processing.
(B) Histogram and Directional FSC plots of RNAPII-nucleosome^{L60}.
These curves were calculated on the 3DFSC server.
(C) The Map-To-Model FSC curve of RNAPII-nucleosome^{L60}.
These curves were calculated using Phenix.

Figure S7

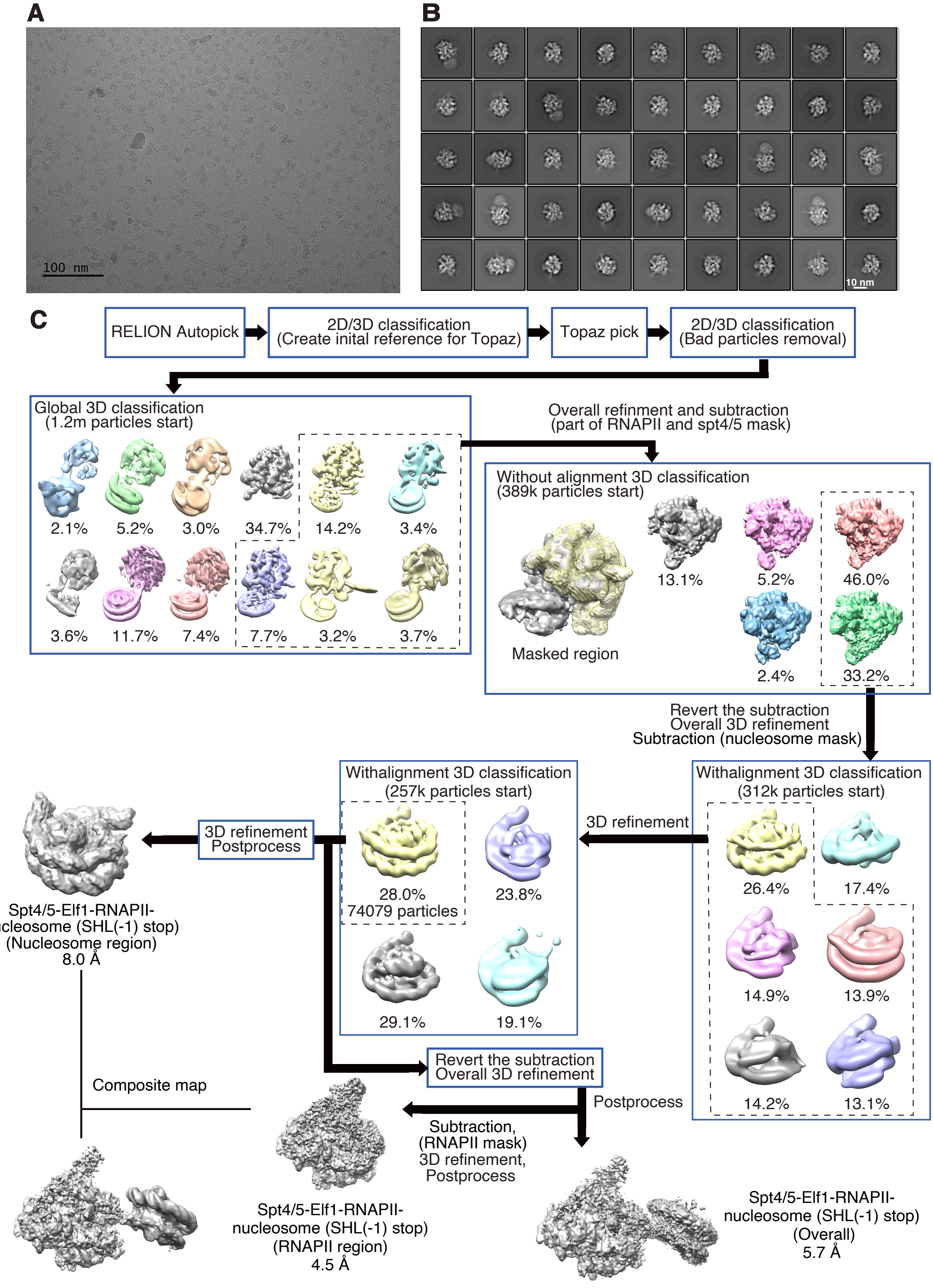

Figure S7: Cryo-EM analysis of the Spt4/5-Elf1-RNAPII-nucleosome complex (SHL(-1) stop).
(A) Representative micrograph of the Spt4/5-Elf1-RNAPII-nucleosome (SHL(-1) stop) dataset.
(B) Representative 2D class averages from the 2D classification after the Topaz picking.
(C) Image processing scheme of the Spt4/5-Elf1-RNAPII-nucleosome (SHL(-1) stop) structure.

Figure S8

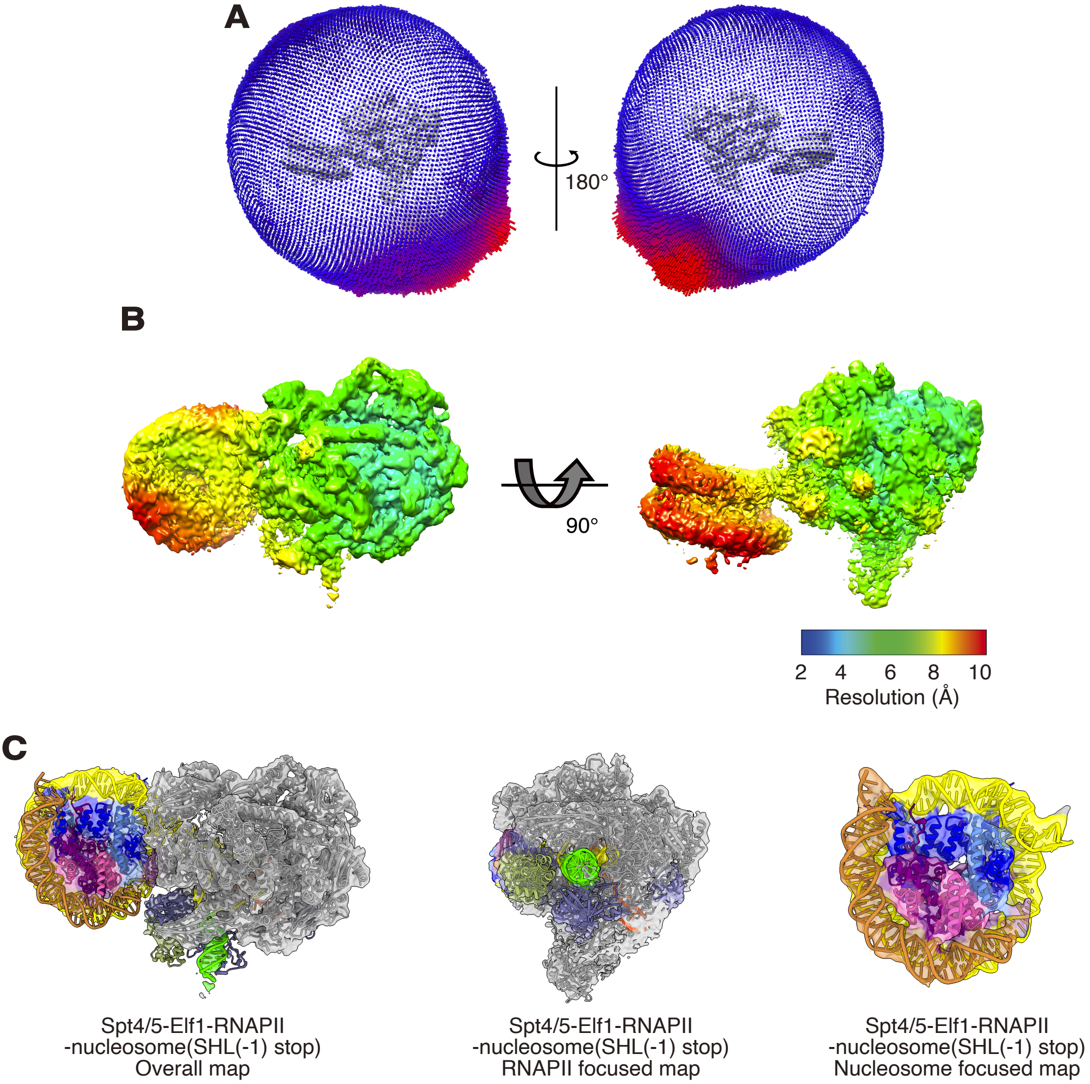

Figure S8: Cryo-EM map qualities of the Spt4/5-Elf1-RNAPII-nucleosome (SHL(-1) stop).
(A) Angular distributions of the Spt4/5-Elf1-RNAPII-nucleosome (SHL(-1) stop).
(B) Local resolution map of the Spt4/5-Elf1-RNAPII-nucleosome (SHL(-1) stop).
(C) Model-to-density fits of the Spt4/5-Elf1-RNAPII-nucleosome (SHL(-1) stop).

Figure S9

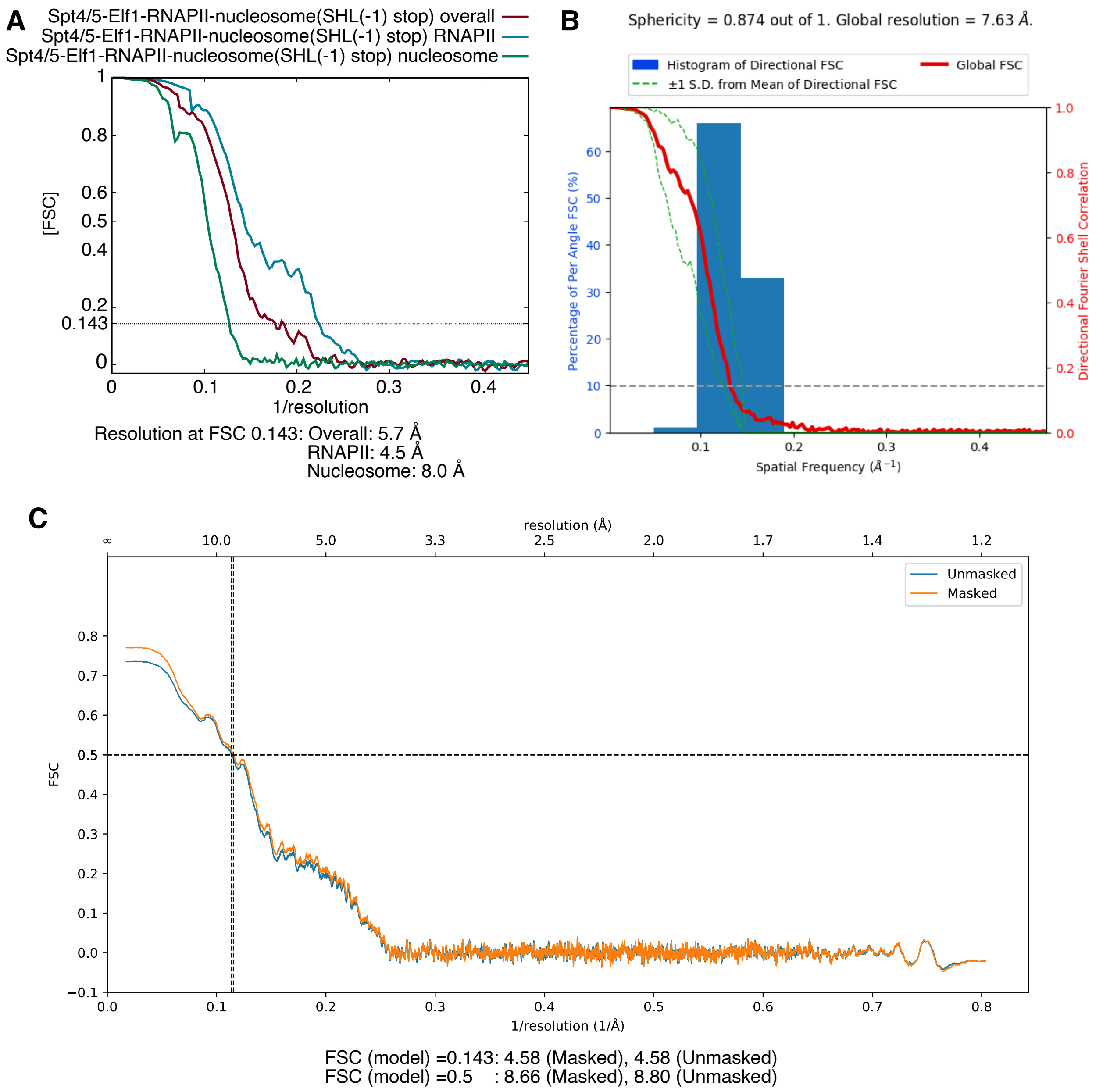

Figure S9: Cryo-EM data qualities of Spt4/5-Elf1-RNAPII-nucleosome (SHL(-1) stop).
(A)Gold-standard Fourier Shell Correlations (FSCs) of the Spt4/5-Elf1-RNAPII nucleosome (SHL(-1) stop). These curves were calculated by RELION post-processing.
(B)Histogram and Directional FSC plots of the Spt4/5-Elf1-RNAPII-nucleosome (SHL(-1) stop). These curves were calculated on the 3DFSC server.
(C)The Map-To-Model FSC curve of the Spt4/5-Elf1-RNAPII-nucleosome (SHL(-1) stop). These curves were calculated using Phenix.

Figure S10

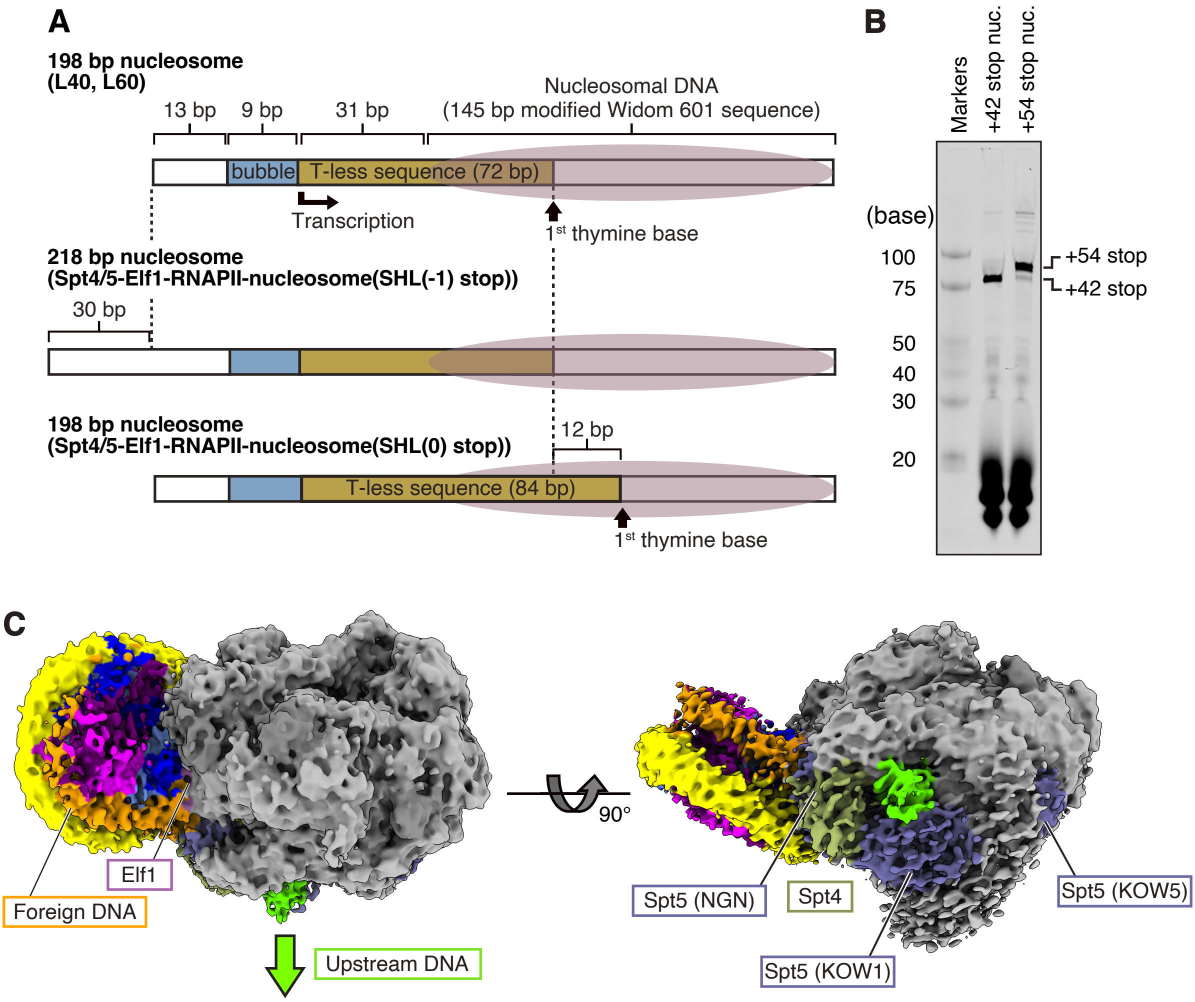

Figure S10: Cryo-EM structure of Spt4/5-Elf1-RNAPII-nucleosome (SHL(0) stop).
(A) Designs of DNA templates for +42 stop and +54 stop nucleosomes.
(B) Denaturing PAGE analysis of +42 stop and +54 stop nucleosome transcription. Bands are detected by DY647 fluorescent dye.
(C) Overall cryo-EM map of the Spt4/5-Elf1-RNAPII-nucleosome (SHL(0) stop). The colors of histones are the same as in Figure 1.

Figure S11

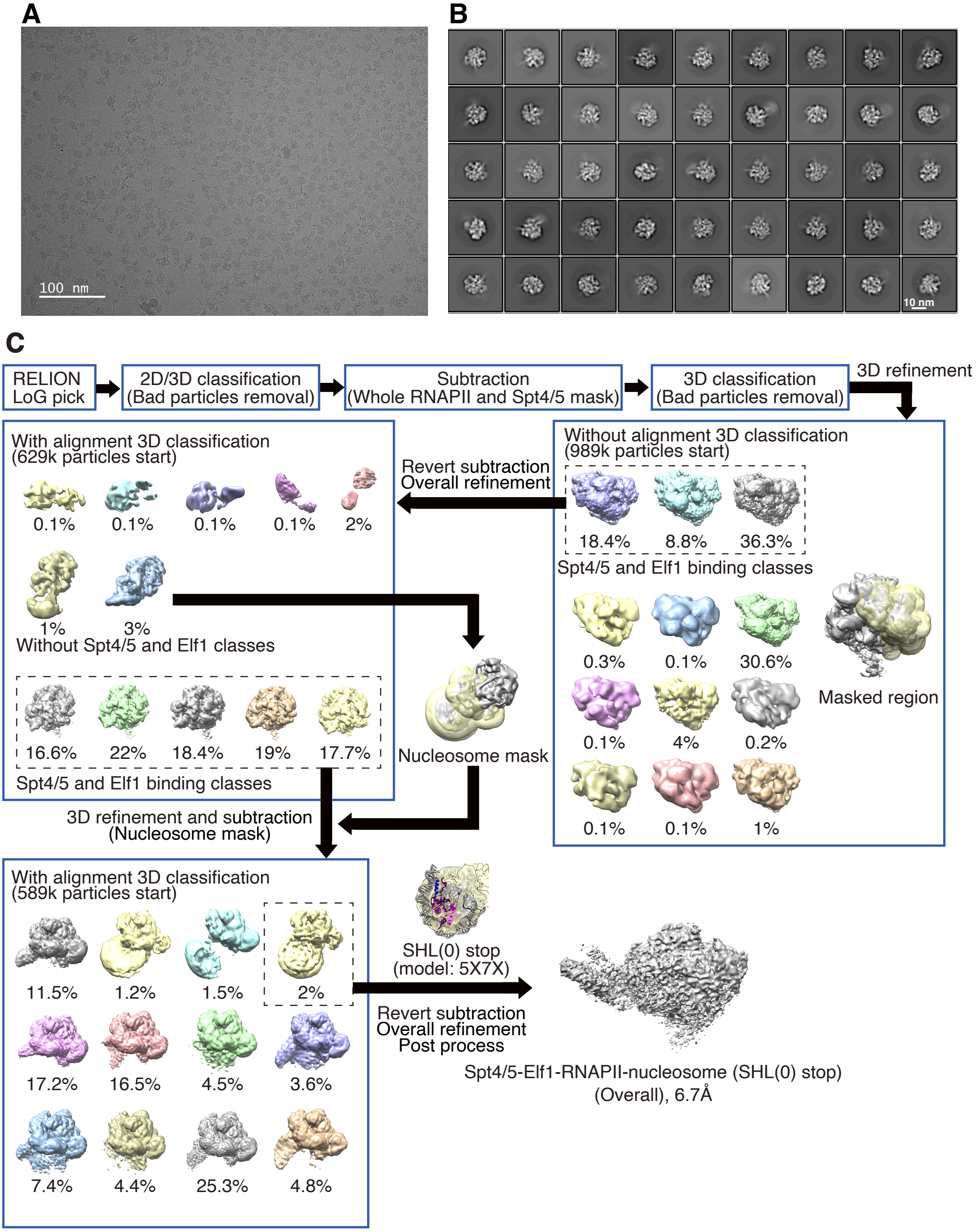

Figure S11: Cryo-EM analysis of the Spt4/5-Elf1-RNAPII-nucleosome (SHL(0) stop).
(A) Representative micrograph of the Spt4/5-Elf1-RNAPII-nucleosome (SHL(0) stop) dataset.
(B) Representative 2D class averages from the 2D classification after the LoG picking.
(C) Image processing scheme of the Spt4/5-Elf1-RNAPII-nucleosome (SHL(0) stop) structure.

Figure S12

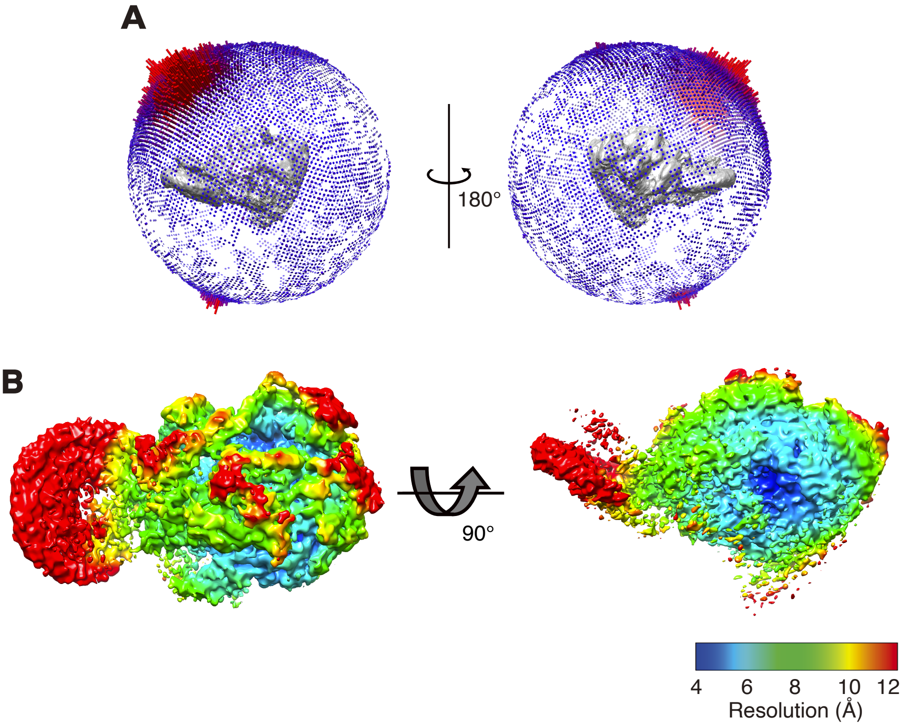

Figure S12: Cryo-EM map qualities of the Spt4/5-Elf1-RNAPII-nucleosome (SHL(0) stop).
(A) Angular distributions of the Spt4/5-Elf1-RNAPII-nucleosome (SHL(0) stop).
(B) Local resolution map of the Spt4/5-Elf1-RNAPII-nucleosome (SHL(0) stop).

Figure S13

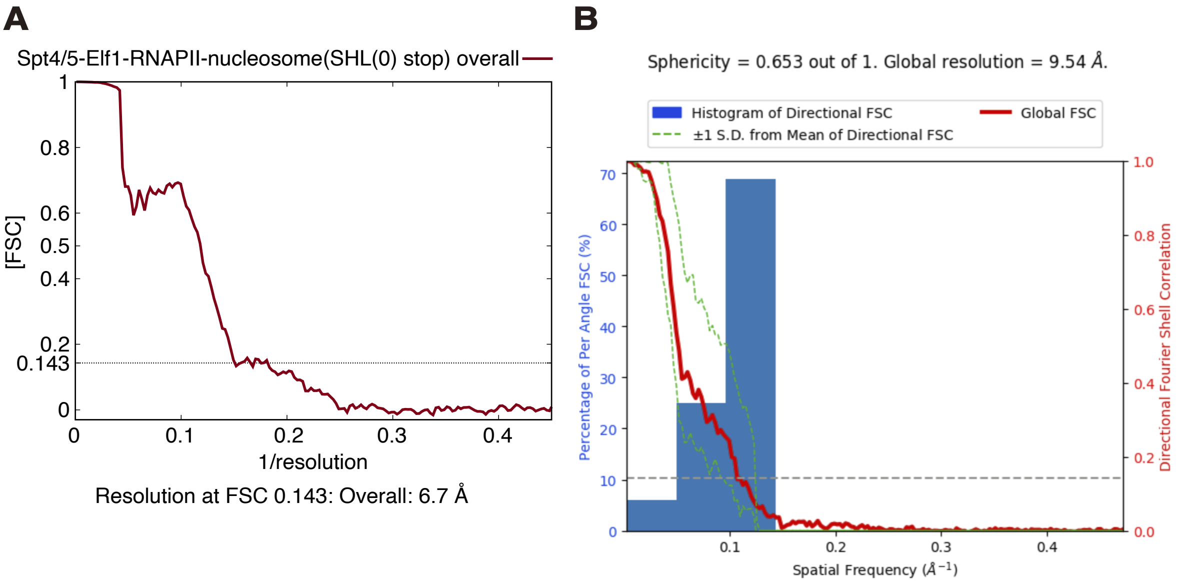

Figure S13: Cryo-EM data qualities of Spt4/5-Elf1-RNAPII-nucleosome (SHL(0) stop).
(A)Gold-standard Fourier Shell Correlations (FSCs) of the Spt4/5-Elf1-RNAPII-nucleosome (SHL(0) stop). These curves were calculated by RELION post-processing.
(B)Histogram and Directional FSC plots of the Spt4/5-Elf1-RNAPII-nucleosome (SHL(0) stop). These curves were calculated on the 3DFSC server.

Figure S14

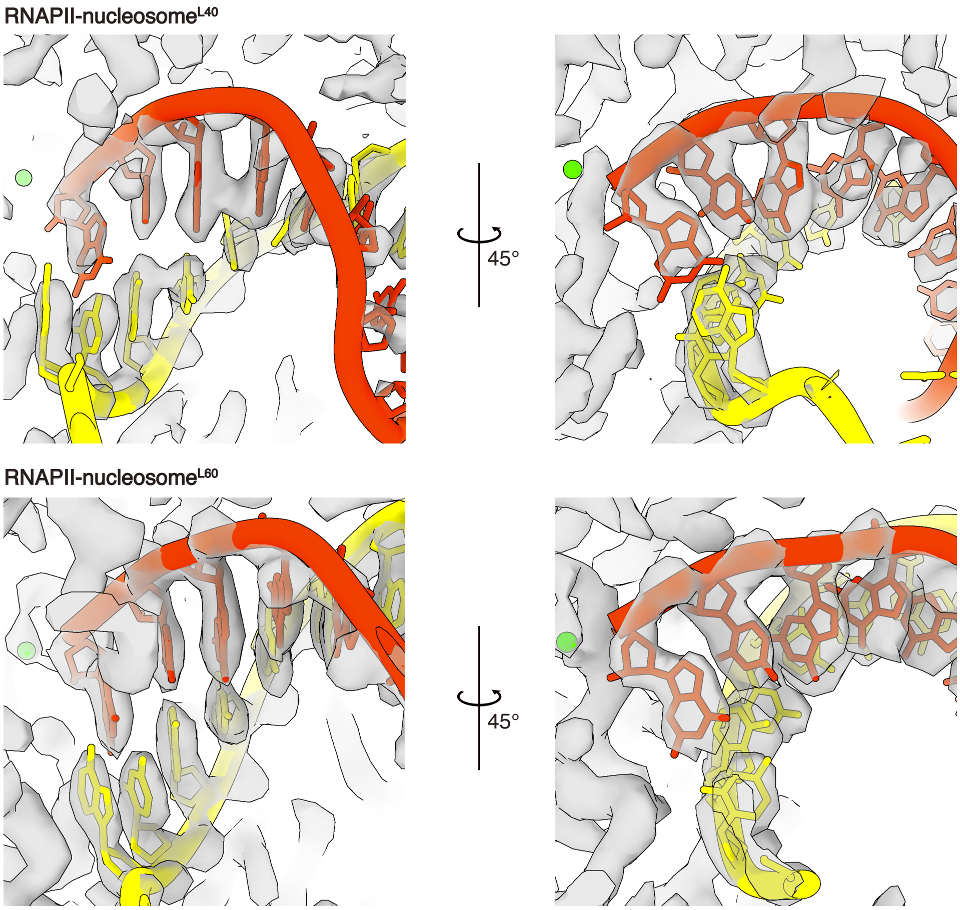

Figure S14: Details of the RNAPII catalytic centers of the models of this study.
The maps are shown in transparent gray. The transcript RNA, template DNA and Mg ion are colored red, yellow and green, respectively.

Figure S15

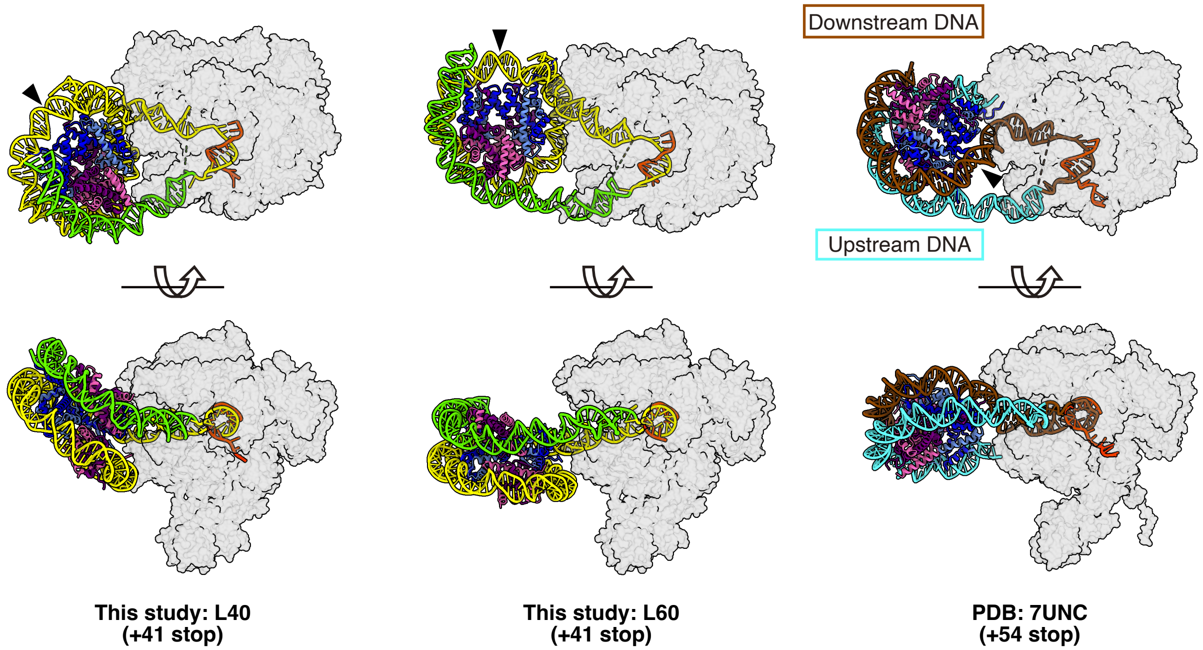

Figure S15: Comparison of the RNAPII-nucleosome complexes with DNA loops.
Structural comparison of the RNAPII-nucleosome^{L40}, the RNAPII-nucleosome^{L60}, and the previous structure of the EC-nucleosome complex paused at the 54 base-pair position (PDB: 7UNC) (19). Upstream DNA is colored green (this study) or cyan (7UNC), and downstream DNA is yellow (this study) or brown (7UNC). The colors of histones are the same as in Figure 1. RNAPII is colored gray, and other elongation factors in 7UNC are not shown. The arrowheads indicate the SHL(0) position.

Table S1: Cryo-EM data collection and image processing.

Sample	RNAPII-nucleosome^{L40}	RNAPII-nucleosome^{L60}	Spt4/5-Elf1-RNAPII-nucleosome (SHL(-1) stop)	Spt4/5-Elf1-RNAPII-nucleosome (SHL(0) stop)
PDB ID	8JH3	8JH4	8JH2	N/A
EMDB ID	EMD-36252	EMD-36253	EMD-36251	EMD-37848
**Data collection**				
Electron microscope	Krios G4 (Thermo Fisher)			
Camera	K3 BioQuantum (Gatan)			
Pixel size (Å/pix)	1.06			
Defocus range (*μ*m)	-1.0 to -2.5			
Number of frames	40			
Dose per frame (e^{-}/Å^{2}/frame)	1.496		1.512	1.567
Number of collected micrographs	9189		11047	17348
Number of selected micrographs	9003		10968	16144
**Reconstruction**				
Number of picked particles	1075376		2308230	4327670
Number of particles used for refinement	51652	97768	74079	12176
Symmetry applied	C1	C1	C1	C1
Final resolution (overall) (Å)	3.7	3.2	5.7	6.7
Final resolution (RNAPII) (Å)	3.3	3.0	4.5	N/A
Final resolution (nucleosome) (Å)	4.1	3.2	8.0	N/A
FSC threshold	0.143			
**Validation**				
MolProbity score	1.68	1.68	1.75	N/A
Clash score	8.81	9.28	5.83	N/A
RMSDs				
Bond lengths (Å)	0.005	0.005	0.003	N/A
Bond angles (°)	0.948	0.971	0.788	N/A
Ramachandran plot (%)				
Outliers	0.07	0.11	0.54	N/A
Allowed	3.20	3.02	6.09	N/A
Favored	96.73	96.87	93.38	N/A
Rotamer outliers (%)	0.35	0.17	0.52	N/A
Q-score	0.255	0.313	0.145	N/A
